# The Manage Care Model – Developing an Evidence-Based and Expert-Driven Chronic Care Management Model for Patients with Diabetes

**DOI:** 10.5334/ijic.4646

**Published:** 2020-04-22

**Authors:** Patrick Timpel, Caroline Lang, Johan Wens, Juan Carlos Contel, Peter E. H. Schwarz, on behalf of the MANAGE CARE Study Group

**Affiliations:** 1 Prevention and Care of Diabetes, Department of Medicine III, Faculty of Medicine Carl Gustav Carus, Technische Universität Dresden, Dresden, DE; 2 Department of General Practice, Faculty of Medicine Carl Gustav Carus, Technische Universität Dresden, Dresden, DE; 3 Department of Primary and Interdisciplinary Care Antwerp, University of Antwerp, Antwerp, BE; 4 Chronic Care Program, Department of Health, Integrated Health and Social Care Plan, Generalitat de Catalunya, ES; 5 Paul Langerhans Institut Dresden, German Center for Diabetes Research (DZD), Dresden, DE

**Keywords:** chronic care, type 2 diabetes mellitus, prevention and health promotion, integrated care, chronic care model

## Abstract

**Background:**

Most current care models are disease- or symptom-focused and mostly do not account for the individual needs of patients with chronic diseases. The aim of this study was to develop an innovative, evidence-based and expert-based practice model for the management of patients with type 2 diabetes mellitus.

**Method:**

An iterative approach was used combining systematic literature search with qualitative methods, including a standardised survey of experts in chronic care (n = 92), an expert workshop of professionals (n = 22) and a multilingual online survey (n = 659). Using three consensus meetings involving researchers, policy makers and experts in chronic care, a limited number of core components and care recommendations was set up to develop a new chronic care model.

**Results:**

The developed ‘MANAGE CARE MODEL’ includes aspects of the health and social care system, resources derived from the living environment, aspects of health promotion and prevention, as well as an expanded understanding of improved outcomes as an integral part of chronic care.

**Conclusion:**

The MANAGE CARE MODEL provides guidance for the development and implementation of chronic care programs, regional networks and national strategies. Future research is needed to validate the model as an instrument of regional chronic care management.

## Background

A growing number of EU citizens suffers from diabetes, posing an emerging health, social and economic burden in the EU [1]. This burden is mostly driven by type 2 diabetes mellitus (T2DM), which is increasingly diagnosed at younger age and leads to a rising number of adults with T2DM aged 65 and older [1]. Due to these rising numbers in diabetes prevalence a growing number of patients faces accompanying comorbidities as well as complex needs [2,3,4,5]. However, many care systems are historically built on separate sectors (health vs. social care, in- vs. outpatient care). This traditional acute and episodic focus of care is inadequate to effectively meet the complex needs of patients as it increases the risk of care fragmentation and loss of information [6,7]. Although these circumstances are repetitively part of health policy initiatives [8,9], there is still an institutional and regulatory separation between health and social care services, as well as between ambulatory and inpatient care [10].

Integrated care is said to improve outcomes of care by linking services of providers along the continuum of care and thus overcoming issues of fragmentation [11]. Back in 1996, Ed Wagner developed the Chronic Care Model (CCM), which became a cornerstone to improve care delivery for chronically ill patients. The CCM comprises the following six components: (1) community, (2) health system, (3) self-management support, (4) delivery system design, (5) decision support and (6) clinical information systems [12]. Ever since, different initiatives developed new models targeting specific weaknesses of the initial CCM.

However, recent analysis uncovered that the understanding of established models’ impact on chronic disease management is limited [13]. This is especially true for the effectiveness and applicability of chronic care models in different populations and settings [13]. Additionally, validated outcome measures, targeting the heterogeneous population of elderly patients, the community they live in as well as their caregivers [14] are needed to improve our understanding of the dynamic and diversified shape of integrated care concepts [15]. Consequently, EU-wide strategies and recommendations for regional implementation of target-group specific chronic care models are still lacking. However, there is a strong need for such guidance as most health systems still (unintentionally) incentivise the treatment of each multiple disorder separately, instead of approaching comorbid patients holistically [16]. Most current care models and treatment guidelines are disease-or symptom-focused and do not include specific instructions on how to prioritise, e.g. diabetes treatment relative to that of other comorbidities and the functional status of patients [13,17].

## Previous work of the MANAGE CARE Study Group

The present study makes use of published results belonging to the same EU-funded project *MANAGE CARE – Active Ageing with Type 2 Diabetes as Model in the Development and Implementation of Innovative Chronic Care Management Models in Europe*. The project included 37 partner institutions like research facilities and national diabetes associations from 17 different countries in Europe.

A **systematic review and meta-analysis** on the effectiveness of the CCM in diabetes care identified eight cluster randomised controlled trials (RCTs) (9,529 patients). However, besides the limited evidence on the effectiveness derived from RCTs and a wide range of outcome measures, only small effects of European multifaceted diabetes care patient outcomes were identified [18].

Additionally, a **standardised survey of experts** in chronic care (n = 92) to analyse existing chronic care programs focusing on effective and missing components, an **expert workshop** (n = 22) to define unmet needs and priorities of elderly patients with T2DM, and a **multilingual online survey** (n = 650) to validate and rank these needs were conducted. Financial support, case management and the consideration of social care were identified as potential areas for improvement in current care models. The expert workshop revealed a number of 150 patient needs, which were grouped into 13 needs dimensions. An online survey was conducted to rank these grouped needs dimensions using an analysis of central tendency (M). *Patient education* was rated as the most important patient need for both patients (M = 4.23, SD = 2.956) and health care professionals (HCP) (M = 4.88, SD = 3.171) in total, followed by *prevention* and *education and knowledge of HCPs* [19].

## Objective

The aim of this study was to develop an innovative, evidence-based and expert-driven practice model for chronic care management of T2DM, addressing the specific needs of patients with diabetes.

## Methods

**Figure 1:**
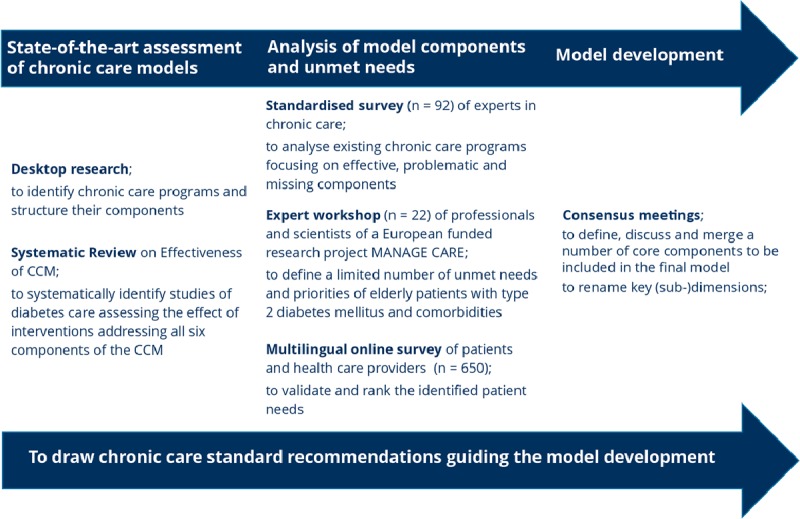
Stepwise development of the model.

An iterative approach combining systematic literature search with qualitative methods and a number of three consensus meeting was used. Figure 1 illustrates the iterative process from gathering data to developing the final model, highlighting the applied methods and their objectives. The process includes steps of previously published analyses and covers the (A) *state-of-the-art assessment*, (B) *analysis of model components and unmet needs*, as well as the (C) *model development*.

### (A) State-of-the-art assessment

A **literature review** was carried out in Medline via PubMed and was complemented by a hand search of global chronic care programs and their components. The search combined long term/chronic care with disease management related terms [Annex 1]. Records were included if they were published after the year 2000, and were written in German or English. Studies were included if they reported on the development of models and programs targeting patients with chronic diseases. Additionally, records were included if they reported on the effectiveness or quality of such models. A standardised protocol was used to summarise relevant information on the identified models. This included aims, target populations, interventions, key stakeholders, features/dimensions and outcomes, which were summarised and structured in a matrix. This matrix was used as a starting point for discussions and expert workshops within the study group (Table 1). Afterwards hand searches were used to identify evidence on the quality and effectiveness of the models and their components.

**Table 1:** Implications and recommendations for the final model.

#	Study design; Objective	Implications and recommendations for the final model

**1**	**Literature review** to identify chronic care programs and structure their components	Rare/**missing data** on the effectiveness and quality of chronic care programs and their components
**2**	**Systematic Review** on effectiveness of CCM [18]; to systematically identify studies of diabetes care assessing the effect of interventions addressing all six components of the CCM	**Limited evidence** on the effectiveness of implementing all CCM-components simultaneously in older patients in Europeand lack of data to understand the intensity of the intervention. Patients with screen-detected T2DM and patients with **newly diagnosed** T2DM showed improved effects on HbA1c → focus on **prevention and health promotion**
**3**	**Standardised survey** (n = 92) of experts in chronic care [19]; to analyse existing chronic care programs focusing on effective, problematic and missing components	“**Financial support**” (no tangible incentives, scarcity of funding, and no refund) regarded as missing in current care programs. **Case management** and quality management should be an integral part of chronic care management. The incorporation of **social services and informal social support**, especially for people with complex health and social care needs, is strongly recommended.
**4**	**Expert workshop** (n = 22) of HCP and experts of a European funded research project MANAGE CARE [19]; to define a limited number of unmet needs and priorities of elderly patients with T2DM and comorbidities	Evidence-based chronic care must be **available and affordable** to patients. **Cooperative systems** are conducive to better chronic care management, including care navigation, care planning and risk stratification. Measures to evaluate the effectiveness, quality and feasibility of careusing **predefined Shared Outcome Frameworks (triple aim)** without being limited to medical outcomes need to be implemented.
**5**	**Multilingual online survey** of patients and health care providers (n = 650) [19]; to validate and rank the identified patient needs	Chronic care must address **individual patient needs** and preferences as much as medical treatment objectives. **Education of patients** as well as **prevention and health promotion** are integral to chronic care management. **Pro-active communication** with the patient should be supported.

The left column shows the five methodological steps and their relevant study objectives combining previously published and current results. The right column summarises recommendations of the single analyses guiding the development of the model.

### (B) Analysis of model components and unmet needs

Previously published data gathered from a **multilingual online survey** (n = 650) to validate and rank unmet needs from the perspective of both patients and health care professionals (HCP) [19] were again evaluated to identify differences in the priorities from the two groups. Mann–Whitney U-test was used to analyse ranks and medians using SPSS for Windows version 23.0 (SPSS, Chicago, IL). Boxplots were used to illustrate the central tendency and spread in those ratings significantly differing between patients and HCPs.

### (C) Model development

The final model was developed following the principles of Stachowiak, indicating that models are a mapping, a reduction and therefore a pragmatic tool [20]. Three **consensus meetings** in February, April and May 2016 were set up to iteratively develop the model using feedback of the MANAGE CARE Study Group. This is in line with previous consensus meetings [21,22,23]. The extensive list of unmet needs was reviewed by the MANAGE CARE Study Group to identify requirements and potential dimensions for the new model. A limited number of core components to be included in the final model was defined, discussed and merged. Three meetings followed a standardised structure. First, the evidence-informed development of the model was described, afterwards the latest changes were presented and subsequently, the current version of the model was reviewed and commented by the participants. The meetings were moderated by one of the authors. As the three meetings were part of workshops of the same project, the participants were slightly changing between the meetings. By combining several feedback loops within the MANAGE CARE Study Group, components of the final model were specified. Renaming of key dimensions and sub-dimensions was supported by further explanations using exemplary application scenarios. These aspects were summarised in a technical handbook describing the developed model and its application. Between the meetings, both attendees and not attending partners were invited to comment on the revised version of the model. During the final meeting, informal consensus was reached.

## Results

### Literature review

The literature review identified 24 health care models from six countries; USA (n = 11), Australia (n = 5), Canada (n = 5), Great Britain (n = 1), Germany (n = 1), and Mexico (n = 1) (annex 2). The expanded version of the chronic care model (eCCM) developed by Barr et al. argues that the CCM is inadequate to proactively cover the heterogeneity and complexities of prevention and health promotion going beyond clinical preventive services [24,25]. Additionally, the authors suggest a stronger focus on supportive communities and public health policies [24]. Other models include the Innovative Care for the Chronic Conditions (ICCC) [26] designed for chronically ill people in low- and middle-income countries, the Improving Chronic Illness Care (ICIC) [27] having a strong focus on integrated practice guidelines, the Stanford Model [28,29,30] designed to enhance regular treatment and disease-specific education in a community setting, and the Transitional Care Model as a nurse-led intervention model [31,32]. Results of the chronic care matrix illustrated a gap in terms of missing evaluations on successful, not successful and missing components of health care models.

### Analysis of the multilingual online-survey

**Figure 2:**
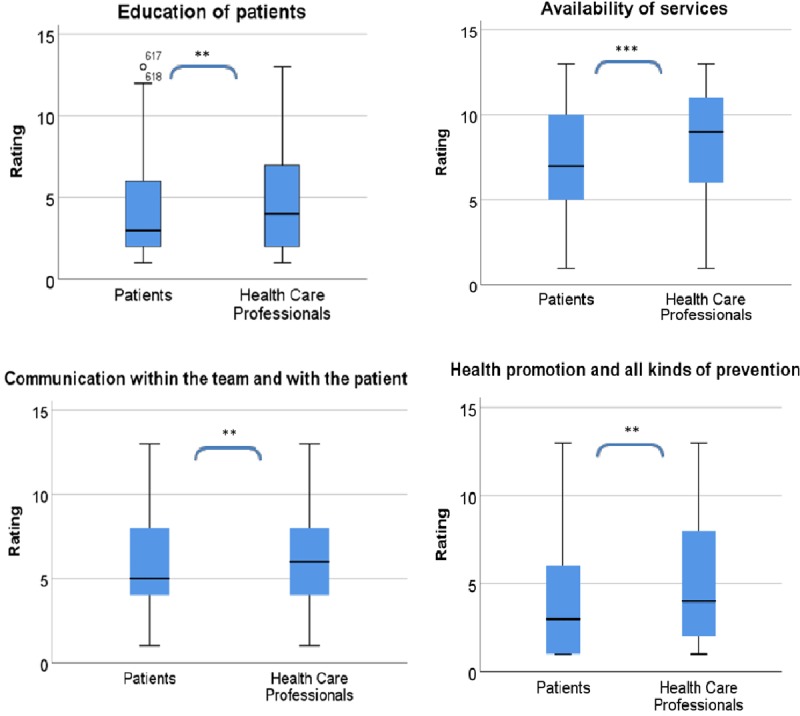
Differences in ratings by user group for selected needs dimensions. The four graphs illustrate the significant (** *p* < 0.01; *** *p* < 0.001) differences in rankings of patients and HCPs for the needs dimensions “Education of patients”, “Health promotion and all kinds of prevention”, “Communication with the team and with the patient” and “Availability of services related to information infrastructure”. All four indicate higher priorities for patients compared to HCPs.

The ranked needs “Education of patients” (M_pat_= 4,23; M_HCP_ = 4.88; *p* < 0.01), “Health promotion and all kinds of prevention” (M_pat_ = 4,28; M_HCP_ = 5.12; *p* < 0.01), “Communication with the team and with the patient” (M_pat_ = 5.59; M_HCP_ = 6.26; *p* < 0.01) and “Availability of services related to information infrastructure” (M_pat_ = 7.29; M_HCP_ = 8.27; *p* < 0.001) are significantly more important to patients compared to HCPs (Figure 2) [19].

### Implications for the model development

Table 1 indicates the implications and recommendations for the final model derived from the iterative process, including the literature review on chronic care programs, the previously published systematic review [18], and the mixed-method approach to identify and rank patients’ needs from different perspectives [19]. The recommendations resulting from the methodological steps 1 to 5 (Table 1) were used as guiding principles to develop the prototype of the model which was later assessed and further developed during three consensus meetings.

### Consensus meetings

The consensus-building process as well as the numbers and backgrounds of their attendees are presented in Table 2. Overall, 30 project partners actively contributed to the consensus meetings. The majority was active in research and academics (n = 18). Employees of a European association (n = 4), national associations (n = 4), a local diabetes educator and a CEO of a regional chronic care network were also participating. Thirteen took part in more than one of the consensus meetings (two meetings n = 7; all three meetings n = 6).

**Table 2:** Description of participants during consensus meetings.

Date, place	Overall number of participants	Number of participants per country

Berlin, February 2016	10	Germany (n = 4); Greece (n = 2); EU (n = 2); Belgium (n = 1); Poland (n = 1)
Athens, April 2016	14	Greece (n = 5); Germany (n = 3); EU (n = 1); Belgium (n = 1); Poland (n = 1); Portugal (n = 1); Lithuania (n = 1); Finland (n = 1)
Barcelona, May 2016	24	Germany (n = 8); Greece (n = 4); Serbia (n = 3); Austria (n = 2); EU (n = 2); Portugal (n = 2); Belgium (n = 1); Lithuania (n = 1); Finland (n = 1)

#### 1^st^ meeting (n = 10)

The following changes were applied after the presentation of the prototype of the model during the first consensus meeting:

- Participants recommended an “active” participation of individuals underlining the proactive orientation of the model with special regard on health promotion and prevention.
- The category “patient” was replaced by “citizen” as healthy individuals and people at disease risk were also considered as relevant for the model.
- The last category “improved outcomes” was recommended to be replaced by “Improved Managed Care”.

#### 2^nd^ meeting (n = 14)

During the second meeting, the participants again called for a stronger focus on prevention and health promotion, as this was one core result of the analysis of patient needs [19]. The adapted target group of “citizens” was considered as too broad. Consequently, the target group of the model was changed to “patients with diabetes (risk)”. Finally, the outcome entities included in the “triple aim” [33] were added to the category of “Improved Managed Care”.

#### 3^rd^ meeting (n = 24)

In the third meeting, a definition of the chronic care teams was considered as useful. Before agreeing on the final model, the category of “Improved Managed Care” was slightly changed to “Improved Integrated Care”. This change was deemed necessary as the model intends to improve quality of chronic care in all three quality domains; structure, process and outcome [34]. As case management is defined as a collaborative process to assess, plan, implement, coordinate, monitor and evaluate the options and services required to meet an individual’s health needs [35], this integration of patient needs was added in the final category. Finally, the participants argued, that although the model targets specific needs of patients with diabetes (risks), the model needs to be applicable in different health systems and settings.

### Description of the overall MANAGE CARE Model

**Figure 3:**
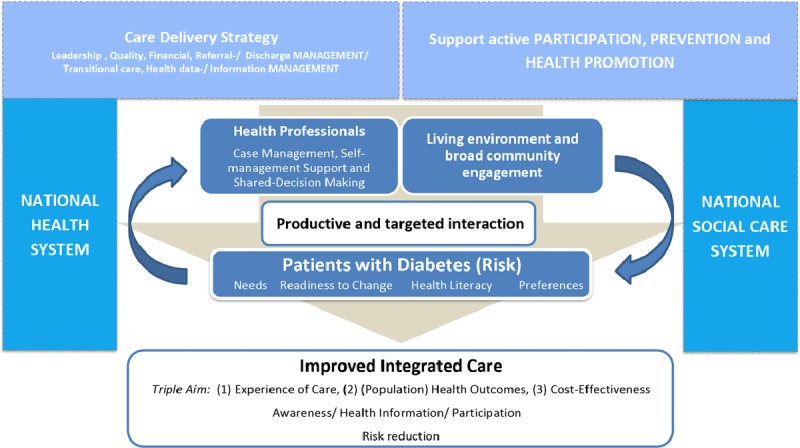
MANAGE CARE Model. MANAGE CARE Model including seven core components for the innovative chronic disease management of patients with diabetes (risk).

Based on the previously published research [18,19], the presented results and the consensus-building process, the MANAGE CARE Study Group decided to include seven dimensions and further sub-categories in the final model (Figure 3), including (1) Care Delivery Strategy, (2) Participation, Prevention and Health Promotion, (3) Health and Social Care System, (4) Health Professionals, (5) Living Environment and Broad Community Engagement, (6) Patients with Diabetes (Risk) and (7) Improved Integrated Care. The MANAGE CARE Model (MCM) is a care-focused model, which incorporates assessments of individual needs to continuously adjust care objectives. According to the salutogenetic focus of the model, an improvement of outcomes is conceptualised by changes in terms of medical, social, lifestyle-related and economical aspects. The model is intended as an orientation framework to guide the development and implementation of regional care models.

#### Description of the model components

##### Care Delivery Strategy: including leadership, quality, financial, referral/discharge/transitional care as well as health data/information management

The MCM advocates a Care Delivery Strategy including six management sub-dimensions enabling responsible clinical, referral/discharge, information, financial and quality management in highly differentiated health systems. There is a growing necessity to balance clinical recommendations with the needs of elderly with T2DM. The development of care delivery strategies should aim to involve multiple disciplines to set up and deliver specialized and preventive care services for people with chronic conditions [36]. To support an effective development and implementation of integrated care pathways, agreements on the whole care continuum are recommended. This includes, among others:

- Diagnosis and referral criteria,
- Responsibility of HCP during stable episodes of the involved patients,
- Preparing for situations of crisis or exacerbation, e.g. by setting up 24/7 coverage schemes to be able to quickly respond in case of acute episodes (especially of patients with multimorbidity),
- Responsibilities and care pathways involving care transitions,
- Coordination with social care services when person has complex social needs.

Care Delivery Strategies are supposed to integrate Information and Communication Technologies (ICT), which facilitate sharing information and virtually linking primary and secondary care (e.g. electronic referrals, shared electronic medical records). Joint commissioning of services should be introduced for common transversal targets between primary and secondary care in order to facilitate integrated care. For example: the rate of 30 day readmissions could be a common transversal indicator where primary and secondary care may be collaboratively accountable [37]. Clinical leadership should be promoted and developed at territories and counties involving them to work together with health authorities constructing integrated care pathways.

##### Support active PARTICIPATION, PREVENTION and HEALTH PROMOTION

The holistic understanding of health and social care characterises the MCM and includes aspects of participation, prevention, health promotion and public health [24,38,39]. This includes both individual level and population-based lifestyle interventions [40,41]. The systematic review by Bongaerts et al. reported a higher potential for newly diagnosed patients [18]. Similarly, other studies suggest that low-risk diabetic patients can benefit from integrated healthcare management programs, including a first health specialist assessment at a diabetes service and a coordinated care approach managed by GPs [42], which is again supporting early detection of patients at risk.

##### National Health and Social Care System

The integration of both the health and social care system is intended to improve the access to vulnerable and high-risk groups of T2DM. This includes social care system coverage of a population through taxes and social charges. It provides guidance for an integrated health and social care system to improve care by combining medical and social needs. The following recommendations may guide this process:

- link the broad community, the patient and the national health as well as social care system for an adequate population-based health planning;
- support collaboration between the health and social care sector as well as between HCPs
- create supportive organizational, governance and leadership structures to achieve a positive and healthy public policy environment
- promote general population stratification incorporating data from hospital health records, primary care minimum data sets, long term care, health care data and social determinants of health
- ensure an optimal health system infrastructure with appropriate availability of health professionals, emergency rooms, hospital beds and alternative day care facilities to manage rapid response in case of crisis
- set up an integrative approach of care including palliative care with hospice-hospital partnerships and long-term care for patients in advanced disease stages
- enhance supportive financing and commissioning of integrated care (e.g. giving local councils and authorities financial support to realize integrative care) and programs targeting uninsured populations.

##### Health Professionals

The MCM is based on an equal participation and collaboration of HCPs, the living environment and the individual citizen. This should be supported by transparent case management, shared-decision making and agreements to jointly develop, carry out and evaluate a productive and targeted interaction. Clinical/professional leadership at the level of counties, regions and municipalities should be supported by collaborators like health authorities to construct regional integrated care pathways. Leaders should be included in the commissioning process (e.g. England – formal “Clinical Commissioning Groups” [CCG]) to support contracts with secondary care.

Chronic disease management, including self-management support for the individual patient, should be carried out by qualified health professionals with adequate experience of care. Their work should be supported by local “integrated clinical/care pathways”, representing a regional agreement of multi-professional experts, clinical leaders and services from both the acute and the primary health care sector. This should also include standards for quality monitoring. Individual care plans appropriate to age, clinical needs, cognitive function, level of frailty, physical level, basic, (psycho-) social and emotional determinants should be developed in collaboration with the patient and informal caregivers. The established phases of case and care management will be used to guide the shared-decision making [43].

##### Living environment and broad community engagement

The MCM uses the full potential and resources of the individual and his living environment (community and informal caregivers). The intention to closely connect the patient and the HCP with the patient environment is based on a strong need to incorporate community-based resources. Supporting local partnerships has the potential to integrate family and neighbourhood as well as communities support in the holistic care of the patient [44,45]. This also strengthens the “living with diabetes” approaches which usually go beyond the self-management of disease-specific symptoms and comorbidities. Additionally, effective local area partnerships with self-help groups, leisure facilities or services supporting the daily living (e.g. transportation to appointments) increase patient autonomy. Such engagement should be culturally appropriate and tailored to the individual needs and priorities of the patient.

##### Patients with Diabetes (Risk) – Assessing Needs, Health Literacy, Preferences

By assessing the individual needs, health risks, health literacy, and preferences, the MCM focusses on individuals at risk, patients with pre-diabetes and manifest T2DM. This individualised perspective enables to include socioeconomic factors, as well as social, psychological and cultural determinants of health and well-being.

##### Productive and targeted interaction

The dynamic relation of the three dimensions, *health professionals, living environment and broad community engagement* and *patients with diabetes (risk)*, is situated in the centre of the MCM. The aim is to provide a productive and targeted interaction. Stratification is applied to classify different levels of needs and priorities of complex diabetic patients. This applies to all aspects of individualised chronic disease management in T2DM patients including diabetes education, self-management support, case management as well as disease management [46,47].

##### Improved Managed Care

The MCM summarises diversified outcomes to evaluate the productive and targeted interaction. It is important to notice that the MCM evaluates more than just medical conditions and goes beyond balancing the *triple aim* of (1) Experience of Care, (2) (Population) Health Outcomes and (3) Cost-Effectiveness [33]. Based on the salutogenetic nature of the model, indicators need to be developed and applied covering aspects of awareness, health information seeking behaviour, participation and reduction of risk factors. *Joint Commissioning of services and Shared Outcome Framework* should be developed by all key stakeholders involved in patient care.

## Discussion

### The developed MANAGE CARE Model

Starting with T2DM as a chronic care example, the MANAGE CARE Study Group developed an innovative chronic care model. This MCM addresses the specific needs of elderly populations being increasingly affected with chronic diseases as well as challenges given by the changes in the health care systems and financing structures in the European countries [8,10].

Both, the literature review and the previously conducted systematic review [18] found only limited evidence on successful, not successful and missing components of the CCM developed by Wagner et al. This is in line with a systematic review on the effectiveness of chronic care models by Davy and colleagues revealing that such models consist of a variety of components. The authors concluded that it was not possible to retrieve which elements or combinations were associated with increased benefits for patients or professionals [48]. Although the CCM defines essential elements which should be implemented to support chronic care patients, it lacks important information on how to handle multimorbidity in the implementation process. They underline that future care models need to account for the dynamic interaction of the model with the everyday life of patients. As such, evaluation of models should consider patient-centered outcomes, the experience of care and resources needed to implement the model [49].

The developed MCM builds on the widely implemented CCM and his expanded version (eCCM) while considering the described weaknesses. While Wagners’ CCM includes the “delivery system design” [12], our MCM changed this element to “care delivery strategy” as the identified evidence and the expert-driven recommendations favour regionally concerted approaches. This includes the identification of relevant disciplines to be involved, defining their responsibilities and jointly pre-defining care pathways as well as quality indicators to be used for the overall chronic care management.

Our MCM also makes use of important elements of the patient-centered medical home model (PCMH), like being team-based, patient-centered, coordinated, and community-oriented [50]. However, the developed MCM puts a stronger emphasis on prevention and supports an active approach to patients at risk for T2DM.

### Discussion of relevant components

#### Health and social care system

The MCM focuses on both the health and the social care system, as this was strongly suggested during the standardised survey and the expert workshop [19]. Increasing staff involvement at the social context level may minimise barriers due to lacking communication and cooperation [51]. This may be accompanied by joint health and social care funds [52]. However, there is a strong need for evidence derived from complex intervention evaluation methodologies in diverse health and social care contexts [53].

#### Targeted interaction

A meta-regression on interventions designed to improve outpatient care of T2DM patients revealed that case management and team changes were associated with the largest pooled reductions in HbA1c values [54]. As disintegrated care originating from HCPs working in isolation and insufficient knowledge of HCPs was identified as a severe limitation of the original CCM [55], we include case and care management delivered by experienced HCPs in our model.

While the CCM is the most evaluated care model, *Self-Management Support* is the most frequent CCM intervention to be associated with statistically significant improvements, predominately for diabetes and hypertension [56]. In addition to self-management support, programs based on collective empowerment in T2DM showed positive effects on clinical as well as behavioural outcomes, and lead to improved self-care [57]. However, Elissenet al. summarise that a better understanding is needed to define what encourages both patients and HCPs to engage in productive interactions [58]. Therefore, a targeted interaction based on integrative and case management approaches was included in the MCM. The MCM also focusses on structured education of patients and HCPs. However, recent analysis uncovered a gap of educational interventions on chronic care for HCP [59].

#### Living environment/community engagement

Barriers derived from a lacking support from family, friends or the community as well as limited considerations of social determinants may impact chronic care outcomes [17]. Therefore, informal care and community engagement were included in the final MCM. Using resources derived from the living environment and the community of patients when implementing the CCM have shown positive results in terms of clinical and behavioural outcomes [26,60]. Similarly, recent evidence suggests that task shifting from physicians to community-based health workers, nurses [61,62] and pharmacists may be of benefit, especially in rural areas [41,63].

#### Improved outcome & Evaluation

Evaluating the benefit of care models is complex and challenging. Yet, the majority of interventions are not sufficiently adapted to the CCM, which is further worsened by a low methodological quality [64]. Studies including balanced measures on multiple performance dimensions are sparse. There is a strong need to consider integrated care settings and their specific context factors [65]. More consistent outcome assessment frameworks are needed. Measurements of outcomes at the health system level is rare, at the population or community level even more missing [14]. Although (informal) caregivers are frequently involved in chronic care, specific outcome measurements targeting family members or friends are missing [14].

The developed MCM uses the triple aim perspective as a starting point for evaluation [33]. In 2012, a fourth dimension – improving the work life of health care providers – was added to also take the changing expectations to primary care systems and regional shortage of primary care services into account [66]. Evidence on general cost efficiencies of integrated care models [67], and their effectiveness carried out in primary as well as social care settings is limited [68]. This is also supported by Busetto et al. calling for intermediate outcome measurements allowing to measure outcomes of specific intervention types [69].

### Future challenges

The developed model is in line with the working definition of “integrated care” as “the management and delivery of health services such that people receive a continuum of health protection, health promotion, disease prevention, diagnosis, treatment, long-term care, rehabilitation and palliative care services through the different levels and sites of care within the health system, and according to their needs” [70]. One important challenge refers to the need for an evaluation of chronic care models in different populations and settings, as there is an unexplained heterogeneity of CCM interventions [71]. As such, future research needs to uncover patient profiles which benefit most from specific interventions, whilst having a stronger emphasis on patients with multiple chronic conditions [72]. On the other hand, HCPs should also be involved in participatory research initiatives in order to include their perceptions on integrated care barriers [7,73,74]. Context, mechanisms and outcomes of multifaceted care needs to be part of future research in diabetes care [18]. Other groups call for more research on the implementation of chronic care models in primary care setting with emphasize on self-efficacy, clinical decision making and organisational capacity [75,76].

Validation studies and evidence focusing on the implementation of the model are needed to gain insights on its applicability and limitations. Both research priorities should take into account the characteristics of the health care systems as well as permutations of mixed systems [77]. This includes country-specific responsibilities of different HCPs (such as hospital-physician relationships or primary care providers as gatekeepers for patients with comorbidities [78]), managed clinical networks, as well as heterogeneous patient populations. This is especially needed, as most evaluation studies originate from the US [13]. A recent analysis of Baptista et al. suggested that implementing single elements of the CCM may not be sufficient to improve clinical outcomes [79]. As such, organisational readiness and an active approach are drivers to achieve improved outcomes [80]. For the MCM, data on the association of single categories, and their overall effects are needed.

This validation process may benefit from recent quality frameworks, such as the Development Model for Integrated Care (DMIC) [81], an improved understanding of linking patients’ quality of life with case management interventions [82] and a more evidence-based analysis of measurements to evaluate integrated care initiatives [83]. Other recently developed models, such as the SELFIE framework for integrated care strongly focus on concepts of multimorbidity grouped at micro-, meso-, and macro levels, which may further guide the validation process [84]. Based on a systematic literature review [85] and intense discussions, the Multimorbidity Care Model was developed by a pan-European collaborative initiative [21]. The authors also call for validation studies in real life settings.

### Strengths and Limitations

One strength of the present study is the iterative combination of different methodological steps, including a previously published systematic review, as well as surveys of experts and patients, and consensus meetings to develop a new model for the treatment of T2DM. That way, an evidence-based as well as expert-driven MCM using T2DM as a starting point was developed. The iterative mixed-method approach, especially the combination of systematic research with different qualitative methods, is another strength of our overall design.

The model has not been validated yet. Theoretical and practical validation studies using different health systems, regions and settings as well as heterogeneous target populations are needed.

## Conclusion

Based on an evidence-based and expert-driven state-of-the-art assessment, knowledge and evidence on existing disease management models and on the needs of elderly people with T2DM was used to develop the MCM. The model has been designed to guide the development and implementation of individualised chronic care models.

Future research is needed to implement and validate the model as an instrument of regional chronic care management.

## Additional Files

The additional files for this article can be found as follows:

10.5334/ijic.4646.s1 Annex 1. List of identified models and programs with countries.

10.5334/ijic.4646.s2 Annex 2. Table 3: Chronic care recommendations derived from a multi-professional expert workshop guiding the final model.

10.5334/ijic.4646.s3 Annex 3. Tables 4 and 5: Dimensions, sub-dimensions and examples derived from the literature review.

## References

[1] International Diabetes Federation. IDF Diabetes Atlas, 7th Edition, Cho N (ed.); 2015 International Diabetes Federation www.diabetesatlas.org.

[2] Iglay K, et al. Prevalence and co-prevalence of comorbidities among patients with type 2 diabetes mellitus. Current Medical Research and Opinion, 2016; 32(7): 243–1252. DOI: 10.1185/03007995.2016.1168291 26986190

[3] Barnett K, et al. Epidemiology of multimorbidity and implications for health care, research, and medical education: a cross-sectional study. The Lancet, 2012; 380(9836): 37–43. DOI: 10.1016/S0140-6736(12)60240-2 22579043

[4] Vos T, et al. Global, regional, and national incidence, prevalence, and years lived with disability for 301 acute and chronic diseases and injuries in 188 countries, 1990–2013: a systematic analysis for the Global Burden of Disease Study 2013. The Lancet, 2015; 386(9995): 743–800. DOI: 10.1016/S0140-6736(15)60692-4 PMC4561509 26063472

[5] Divo MJ, Martinez CH, Mannino DM. Ageing and the epidemiology of multimorbidity. The European respiratory journal, 2014; 44(4): 1055–1068. DOI: 10.1183/09031936.00059814 25142482 PMC4918092

[6] Amelung V, Hildebrandt H, Wolf S. Integrated care in Germany—a stony but necessary road! International Journal of Integrated Care, 2012; 12: e16 DOI: 10.5334/ijic.853 22977429 PMC3429136

[7] Lang C, et al. “Da kann man sich ja totklingeln, geht ja keiner ran” – Schnittstellenprobleme zwischen stationärer, hausärztlicher und ambulant-fachspezialisierter Patientenversorgung aus Sicht Dresdner Hausärzte. Gesundheitswesen, 2019; 81(10): 822–830. DOI: 10.1055/a-0664-0470 30114720

[8] Ezeh AC, Bongaarts J, Mberu B. Global population trends and policy options. The Lancet, 2012; 380(9837): 142–148. DOI: 10.1016/S0140-6736(12)60696-5 22784532

[9] Erler A, Fullerton B, Nolte E. Germany, in Assessing Chronic Disease Management in European Health Systems: Country reports Nolte E, Knai C (eds.), European Observatory on Health Systems and Policies. Copenhagen, Denmark; 2015. 29035490

[10] Busse R, Blümel M. Germany: health system review, in Health Systems in Transition. Busse R (ed.), European Observatory on Health Systems and Policies; 2014 1–296. 25115137

[11] Expert Group on Health Systems Performance Assessment. BLOCKS – Tools and Methodologies to Assess Integrated Care in Europe, European Commission (ed.). Luxembourg: Publications Office of the European Union; 2017.

[12] Wagner EH, Austin BT, Von Korff M. Organizing care for patients with chronic illness. Milbank Q, 1996; 74(4): 511–44. DOI: 10.2307/3350391 8941260

[13] Grover A, Joshi A. An overview of chronic disease models: a systematic literature review. Glob J Health Sci, 2015; 7(2): 210–227. DOI: 10.5539/gjhs.v7n2p210 PMC4796376 25716407

[14] Drouin H, et al. Measured outcomes of chronic care programs for older adults: a systematic review. BMC Geriatrics, 2015; 15: 139 DOI: 10.1186/s12877-015-0136-7 26503159 PMC4621859

[15] Armitage GD, et al. Health systems integration: state of the evidence. International Journal of Integrated Care, 2009; 9: e82 DOI: 10.5334/ijic.316 19590762 PMC2707589

[16] Clarke JL, et al. An Innovative Approach to Health Care Delivery for Patients with Chronic Conditions. Population Health Management, 2017; 20(1): 23–30. DOI: 10.1089/pop.2016.0076 27563751 PMC5278805

[17] Siminerio LM, Piatt G, Zgibor JC. Implementing the chronic care model for improvements in diabetes care and education in a rural primary care practice. Diabetes Educ, 2005; 31(2): 225–234. DOI: 10.1177/0145721705275325 15797851

[18] Bongaerts BWC, et al. Effectiveness of chronic care models for the management of type 2 diabetes mellitus in Europe: a systematic review and meta-analysis. BMJ Open, 2017; 7(3): 1–16. DOI: 10.1136/bmjopen-2016-013076 PMC5372084 28320788

[19] Timpel P, et al. Individualising Chronic Care Management by Analysing Patients’ Needs – A Mixed Method Approach. International Journal of Integrated Care, 2017; 17(6): 12 DOI: 10.5334/ijic.3067 29588635 PMC5854149

[20] Stachowiak H. Allgemeine Modelltheorie, 1973 Springer-Verlag, Wien DOI: 10.1007/978-3-7091-8327-4

[21] Palmer K, et al. Multimorbidity care model: Recommendations from the consensus meeting of the Joint Action on Chronic Diseases and Promoting Healthy Ageing across the Life Cycle (JA-CHRODIS). Health Policy, 2018; 122(1): 4–11. DOI: 10.1016/j.healthpol.2017.09.006 28967492

[22] Corazza GR, et al. A consensus for the development of a vector model to assess clinical complexity. Internal and Emergency Medicine, 2017; 12(8): 1313–1318. DOI: 10.1007/s11739-017-1709-6 28710713

[23] Ewert B, et al. Building a taxonomy of integrated palliative care initiatives: results from a focus group. BMJ supportive & palliative care, 2016; 6(1): 14–20. DOI: 10.1136/bmjspcare-2014-000841 PMC4789694 26647043

[24] Barr VJ, et al. The expanded Chronic Care Model: an integration of concepts and strategies from population health promotion and the Chronic Care Model. Hosp Q, 2003; 7(1): 73–82. DOI: 10.12927/hcq.2003.16763 14674182

[25] Frei A, et al. The Chronic CARe for diAbeTes study (CARAT): a cluster randomized controlled trial. Cardiovascular Diabetology, 2010; 9: 23–23. DOI: 10.1186/1475-2840-9-23 20550650 PMC2902433

[26] Nuno R, et al. Integrated care for chronic conditions: the contribution of the ICCC Framework. Health Policy, 2012; 105(1): 55–64. DOI: 10.1016/j.healthpol.2011.10.006 22071454

[27] Group Health Research Institute. Improving Chronic Illness Care. 2006–2015. 2015, [03.09.2015]; Available from: http://www.improvingchroniccare.org/.

[28] Lorig KR, et al. Chronic Disease Self-Management Program: 2-Year Health Status and Health Care Utilization Outcomes. Medical Care, 2001; 39(11): 1217–1223. DOI: 10.1097/00005650-200111000-00008 11606875

[29] Lorig KR, et al. Evidence Suggesting That a Chronic Disease Self-Management Program Can Improve Health Status While Reducing Hospitalization: A Randomized Trial. Medical Care, 1999; 37(1): 5–14. DOI: 10.1097/00005650-199901000-00003 10413387

[30] Stanford Patient Education Research Center. Chronic Disease Self-Management Program. 2015 [04.09.2015]; Available from: http://patienteducation.stanford.edu/programs/cdsmp.html.

[31] Naylor MD, et al. Transitional care of older adults hospitalized with heart failure: a randomized, controlled trial. J Am Geriatr Soc, 2004; 52(5): 675–84. DOI: 10.1111/j.1532-5415.2004.52202.x 15086645

[32] Hirschman KB, et al. Continuity of Care: The Transitional Care Model. OJIN: The Online Journal of Issues in Nursing, 2015; 20(3). 26882510

[33] Berwick DM, Nolan TW, Whittington J. The Triple Aim: Care, Health, And Cost. Health Affairs, 2008; 27(3): 759–769. DOI: 10.1377/hlthaff.27.3.759 18474969

[34] Donabedian A. Evaluating the Quality of Medical Care. The Milbank Quarterly, 2005; 83(4): 691–729. DOI: 10.1111/j.1468-0009.2005.00397.x 16279964 PMC2690293

[35] Case Management Society of America. What Is A Case Manager? 2017 [accessed 2019 07 – 06]; Available from: http://www.cmsa.org/who-we-are/what-is-a-case-manager/.

[36] Rumball-Smith J, et al. Under the same roof: co-location of practitioners within primary care is associated with specialized chronic care management. BMC Family Practice, 2014; 15: 149 DOI: 10.1186/1471-2296-15-149 25183554 PMC4171578

[37] Drincic A, et al. The effect of diabetes case management and Diabetes Resource Nurse program on readmissions of patients with diabetes mellitus. Journal of clinical & translational endocrinology, 2017; 8: 29–34. DOI: 10.1016/j.jcte.2017.03.003 29067256 PMC5651336

[38] Mueller G, Weser G, Schwarz PEH. The European perspective of diabetes prevention: The need for individualization of diabetes prevention. Journal of Endocrinological Investigation, 2013; 36(5): 352–357. DOI: 10.1007/BF03347104 23712196

[39] Lemmens LC, et al. Patient involvement in diabetes care: experiences in nine diabetes care groups. International Journal of Integrated Care, 2015; 15: e044 DOI: 10.5334/ijic.2207 27118961 PMC4843182

[40] Greaves CJ, et al. Systematic review of reviews of intervention components associated with increased effectiveness in dietary and physical activity interventions. BMC Public Health, 2011; 11: 1–12. DOI: 10.1186/1471-2458-11-119 21333011 PMC3048531

[41] Schwarz PEH, et al. Blood Sugar Regulation for Cardiovascular Health Promotion and Disease Prevention: JACC Health Promotion Series. Journal of the American College of Cardiology, 2018; 72(15): 1829–1844. DOI: 10.1016/j.jacc.2018.07.081 30286928 PMC6709577

[42] Baldo V, et al. Diabetes outcomes within integrated healthcare management programs. Primary Care Diabetes, 2017; 9(1): 54–59. DOI: 10.1016/j.pcd.2014.03.005 24746417

[43] Lukersmith S, Millington M, Salvador-Carulla L. What Is Case Management? A Scoping and Mapping Review. International journal of integrated care, 2016; 16(4): 2–2. DOI: 10.5334/ijic.2477 PMC5388031 28413368

[44] Gimpel N, et al. Patient perceptions of a community-based care coordination system. Health Promot Pract, 2010; 11(2): 173–81. DOI: 10.1177/1524839908320360 19131540

[45] Philis-Tsimikas A, et al. Improvement in Diabetes Care of Underinsured Patients Enrolled in Project Dulce. Diabetes Care, 2004; 27(1): 110 DOI: 10.2337/diacare.27.1.110 14693975

[46] Zeng Z, et al. Effect of case management on patients with type 2 diabetes mellitus: a meta-analysis. Chinese Nursing Research, 2016; 3(2): 71–76. DOI: 10.1016/j.cnre.2016.06.008

[47] Pfeiffer AFH, Klein HH. The Treatment of Type 2 Diabetes. Dtsch Arztebl International, 2014; 111(5): 69–82. 10.3238/arztebl.2014.0069 PMC3952010 24612534

[48] Davy C, et al. Effectiveness of chronic care models: opportunities for improving healthcare practice and health outcomes: a systematic review. BMC Health Serv Res, 2015; 15: 194 DOI: 10.1186/s12913-015-0854-8 25958128 PMC4448852

[49] Boehmer KR, et al. Does the chronic care model meet the emerging needs of people living with multimorbidity? A systematic review and thematic synthesis. PloS One, 2018; 13(2): e0190852 DOI: 10.1371/journal.pone.0190852 29420543 PMC5805171

[50] Jackson GL, et al. The Patient-Centered Medical Home: A Systematic Review. Annals of Internal Medicine, 2013; 158(3): 169–178. DOI: 10.7326/0003-4819-158-3-201302050-00579 24779044

[51] Busetto L, et al. Context, mechanisms and outcomes of integrated care for diabetes mellitus type 2: a systematic review. BMC Health Serv Res, 2016; 16: 18 DOI: 10.1186/s12913-015-1231-3 26772769 PMC4715325

[52] Allen K, Glasby J, Rodrigues R. Joint Working between Health and Social Care In Long-Term Care in Europe: Improving Policy and Practice, Leichsenring K, Billings J, Nies H (eds.). New York: Palgrave Macmillan; 2013 81–99. DOI: 10.1057/9781137032348_4

[53] Hanlon P, et al. A systematic review of interventions by healthcare professionals to improve management of non-communicable diseases and communicable diseases requiring long-term care in adults who are homeless. BMJ Open, 2018; 8(4): e020161 DOI: 10.1136/bmjopen-2017-020161 PMC5892758 29627814

[54] Shojania KG, et al. Effects of quality improvement strategies for type 2 diabetes on glycemic control: A meta-regression analysis. JAMA, 2006; 296(4): 427–440. DOI: 10.1001/jama.296.4.427 16868301

[55] Yeoh EK, et al. Benefits and limitations of implementing Chronic Care Model (CCM) in primary care programs: A systematic review. International Journal of Cardiology, 2018; 258: 279–288. DOI: 10.1016/j.ijcard.2017.11.057 29544944

[56] Reynolds R, et al. A systematic review of chronic disease management interventions in primary care. BMC Fam Pract, 2018; 19(1): 11 DOI: 10.1186/s12875-017-0692-3 29316889 PMC5759778

[57] Baldoni NR, et al. Collective empowerment strategies for patients with Diabetes Mellitus: A systematic review and meta-analysis. Primary Care Diabetes, 2017; 11(2): 201–211. DOI: 10.1016/j.pcd.2016.09.006 27780683

[58] Elissen A, et al. Is Europe putting theory into practice? A qualitative study of the level of self-management support in chronic care management approaches. BMC Health Services Research, 2013; 13: 117–117. DOI: 10.1186/1472-6963-13-117 23530744 PMC3621080

[59] Bogetz JF, et al. Training Health Care Professionals for 21st-Century Practice: A Systematic Review of Educational Interventions on Chronic Care. Academic Medicine, 2015; 90(11): 1561–1572. DOI: 10.1097/ACM.0000000000000773 26039140

[60] Piatt GA, et al. Translating the chronic care model into the community: results from a randomized controlled trial of a multifaceted diabetes care intervention. Diabetes Care, 2006; 29(4): 811–7. DOI: 10.2337/diacare.29.04.06.dc05-1785 16567820

[61] Welch G, et al. Nurse diabetes case management interventions and blood glucose control: results of a meta-analysis. Diabetes Res Clin Pract, 2010; 88(1): 1–6. DOI: 10.1016/j.diabres.2009.12.026 20116879

[62] Seidu S, et al. A systematic review of interventions targeting primary care or community based professionals on cardio-metabolic risk factor control in people with diabetes. Diabetes Res Clin Pract, 2016; 113: 1–13. DOI: 10.1016/j.diabres.2016.01.022 26972954

[63] Jeet G, et al. Community health workers for non-communicable diseases prevention and control in developing countries: Evidence and implications. PLoS One, 2017; 12(7): e0180640 DOI: 10.1371/journal.pone.0180640 28704405 PMC5509237

[64] Seitz P, et al. Interventions in primary care to improve cardiovascular risk factors and glycated haemoglobin (HbA1c) levels in patients with diabetes: a systematic review. Diabetes Obes Metab, 2011; 13(6): 479–89. DOI: 10.1111/j.1463-1326.2010.01347.x 21205119

[65] Minkman M, Ahaus K, Huijsman R. Performance improvement based on integrated quality management models: what evidence do we have? A systematic literature review. Int J Qual Health Care, 2007; 19(2): 90–104. DOI: 10.1093/intqhc/mzl071 17277010

[66] Bodenheimer T, Sinsky C. From Triple to Quadruple Aim: Care of the Patient Requires Care of the Provider. The Annals of Family Medicine, 2014; 12(6): 573–576. DOI: 10.1370/afm.1713 25384822 PMC4226781

[67] Desmedt M, et al. Economic Impact of Integrated Care Models for Patients with Chronic Diseases: A Systematic Review. Value Health, 2016; 19(6): 892–902. DOI: 10.1016/j.jval.2016.05.001 27712719

[68] Damery S, Flanagan S, Combes G. Does integrated care reduce hospital activity for patients with chronic diseases? An umbrella review of systematic reviews. BMJ Open, 2016; 6(11): e011952 DOI: 10.1136/bmjopen-2016-011952 PMC5129137 27872113

[69] Busetto L, et al. Intervention types and outcomes of integrated care for diabetes mellitus type 2: a systematic review. J Eval Clin Pract, 2016; 22(3): 299–310. DOI: 10.1111/jep.12478 26640132

[70] Kluge H. Developing a regional action framework for coordinated/integrated Health Services Delivery (CIHSD) in the WHO European region. International Journal of Integrated Care. 2013; 13(6): None DOI: 10.5334/ijic.1328 PMC3886596 24409110

[71] Drewes HW, et al. The effectiveness of chronic care management for heart failure: meta-regression analyses to explain the heterogeneity in outcomes. Health Serv Res, 2012; 47(5): 1926–59. DOI: 10.1111/j.1475-6773.2012.01396.x 22417281 PMC3513612

[72] de Bruin SR, et al. Comprehensive care programs for patients with multiple chronic conditions: a systematic literature review. Health Policy, 2012; 107(2–3): 108–45. DOI: 10.1016/j.healthpol.2012.06.006 22884086

[73] Harris J, et al. How patient and community involvement in diabetes research influences health outcomes: A realist review. Health expectations: an international journal of public participation in health care and health policy, 2019; 22(5): 907–920. DOI: 10.1111/hex.12935 31286639 PMC6803418

[74] Sarrami-Foroushani P, et al. Implementing strategies in consumer and community engagement in health care: results of a large-scale, scoping meta-review. BMC health services research, 2014; 14: 402–402. DOI: 10.1186/1472-6963-14-402 25230846 PMC4177168

[75] Stellefson M, Dipnarine K, Stopka C. The chronic care model and diabetes management in US primary care settings: a systematic review. Prev Chronic Dis, 2013; 10: E26 DOI: 10.5888/pcd10.120180 23428085 PMC3604796

[76] Kadu MK, Stolee P. Facilitators and barriers of implementing the chronic care model in primary care: a systematic review. BMC Fam Pract, 2015; 16: 12 DOI: 10.1186/s12875-014-0219-0 25655401 PMC4340610

[77] Nolte E, Knai C, Saltman RB. Assessing chronic disease management in European health systems Concepts and Approaches. Copenhagen, Denmark: European Observatory of Health Systems and Policies; 2014. 29019637

[78] Hofmarcher MM, Oxley H, Rusticelli E. Improved Health System Performance Through Better Care Coordination In OECD Health Working Paper No. 30, O.H.W. Papers (ed.) Paris, France; 2007 86.

[79] Baptista DR, et al. The chronic care model for type 2 diabetes: a systematic review. Diabetol Metab Syndr, 2016; 8: 7 DOI: 10.1186/s13098-015-0119-z 26807158 PMC4722715

[80] Kampstra NA, et al. Health outcomes measurement and organizational readiness support quality improvement: a systematic review. BMC health services research, 2018; 18(1): 1005–1005. DOI: 10.1186/s12913-018-3828-9 30594193 PMC6311059

[81] Mirella M. The Development Model for Integrated Care: a validated tool for evaluation and development. Journal of Integrated Care, 2016; 24(1): 38–52. DOI: 10.1108/JICA-01-2016-0005

[82] Flanagan S, Damery S, Combes G. The effectiveness of integrated care interventions in improving patient quality of life (QoL) for patients with chronic conditions. An overview of the systematic review evidence. Health Qual Life Outcomes, 2017; 15(1): 188 DOI: 10.1186/s12955-017-0765-y 28962570 PMC5622519

[83] Bautista MAC. et al. Instruments Measuring Integrated Care: A Systematic Review of Measurement Properties. The Milbank Quarterly, 2016; 94(4): 862–917. DOI: 10.1111/1468-0009.12233 27995711 PMC5192798

[84] Leijten FRM, et al. The SELFIE framework for integrated care for multi-morbidity: Development and description. Health Policy, 2018; 122(1): 12–22. DOI: 10.1016/j.healthpol.2017.06.002 28668222

[85] Hopman P, et al. Effectiveness of comprehensive care programs for patients with multiple chronic conditions or frailty: A systematic literature review. Health Policy, 2016; 120(7): 818–832. DOI: 10.1016/j.healthpol.2016.04.002 27114104

