# Supplementary material for: The Manage Care Model – Developing an Evidence-Based and Expert-Driven Chronic Care Management Model for Patients with Diabetes
Source: Int J Integr Care. 2020 Apr 22;20(2):2. doi: 10.5334/ijic.4646 (PMC7181948; doi:10.5334/ijic.4646)
Supplement: Annex 1. — List of identified models and programs with countries. [file ijic-20-2-4646-s1.pdf]

Annex 1 – List of identified models and programs with countries

UNITED STATES

- Chronic Care Model
- Improving Chronic Illness Care (ICIC)
- Innovative Care for Chronic Conditions (ICCC)
- The Faces of Medicaid II: Recognizing the Care Needs of People with Multiple Chronic Conditions
- Population Management for Chronic Conditions
- Medical Home
- Care for chronic non-communicable diseases
- Patient-Centered Medical Home (PCMH)
- Stanford Model
- Community-based Transition Model
- Transitional Care Model

CANADA

- Expanded Chronic Care Model (eCCM)
- Transforming Care for Canadians with chronic health conditions
- Chronic Illness Care Management
- Preventing and Managing Chronic Disease: Ontario's Framework
- Chronic Disease Management

UNITED KINGDOM

- The English NHS and Social Care Long Term Conditions Model

AUSTRALIA

- Improving health care for people with chronic illness: a blueprint for change 2001-2003
- NSW Chronic Care Program Phase One: 2000-2003
- NSW Chronic Care Program Phase Two: 2003-2006
- NSW Chronic Care Program Phase Three: 2006-2009
- Chronic Care for Aboriginal People (CCAP)

GERMANY

- Gesundes Kinzigtal

MEXICO

- Veracruz Initiative for Diabetes Awareness (VIDA)
