# Supplementary material for: The Manage Care Model – Developing an Evidence-Based and Expert-Driven Chronic Care Management Model for Patients with Diabetes
Source: Int J Integr Care. 2020 Apr 22;20(2):2. doi: 10.5334/ijic.4646 (PMC7181948; doi:10.5334/ijic.4646)
Supplement: Annex 2. — Table 3: Chronic care recommendations derived from a multi-professional expert workshop guiding the final model. [file ijic-20-2-4646-s2.pdf]

Annex 2

Table 3 Chronic care recommendations derived from a multi-professional expert workshop guiding the final model

**1**	**Education**	Continuing literacy and need-adjusted education is essential for both patients and health care providers. Promote and apply diabetes self-management care through patient education, empowerment and shared-decision making. Patient motivation support for lifestyle behavioural change and their compliance to therapy should be an integral component of care.
**2**	**Individual needs**	Chronic care must address individual patient needs and preferences as much as medical treatment objectives. Patient needs, risks and comorbidities have to be assessed, addressed and regularly monitored.
**3**	**Prevention & Health Promotion**	Prevention activities are integral to chronic care management. Activities must be measurable, sustainable and needs oriented. Contribute actively to prevention focusing on target groups. Prevention products must primarily address individual needs.<br>Contribute actively to awareness campaigns and health promotion focusing on target groups. Provide tools which primarily address individual needs for preventive education and empowerment. Provide the most effective policy action to cater for screening and early detection.
**4**	**Social support & Community Engagement**	A supportive social environment must accompany chronic patients at all levels, settings and phases of care. One peer or counterpart is the minimum to provide this support.<br>Create and support community care approaches to improve care access of vulnerable target groups (minorities, disabled, etc.).
**5**	**Accessibility**	Evidence-based chronic care must be available and affordable to patients. Provide easy and timely access to care. Ensure continuity between different levels of health system and involve municipalities in prevention and treatment activities. Access must include 24/7 minimum services for crisis and exacerbation, especially for patients with diabetes and other co-morbidities who could be in a complex condition with health and potential social care needs.
**6**	**Cooperation/ coordination**	Cooperative systems are conducive to better chronic care management, including care navigation, care planning and risk stratification. Promote multidisciplinary cooperation and exchange between health care providers, formal and informal caregivers around primary care by data exchange and quarterly meetings. Different kinds of instrumental operative items should be implemented to facilitate and guarantee a coordinated and integrated care environment at local level.
**7**	**Sharing information**	Personal data is owned by the patients. Anonymised patient data are a public health asset for policy-making. All stakeholders relevant to patient care must have timely access to patient data. Integrate individualised shared decision making, promote cooperation and exchange between health care providers, formal and informal caregivers by data exchange and quarterly meetings. This data could be used to facilitate good calculation of indicators (diabetes related and management related like hospital rates utilisation, etc).<br>This aggregate information derived from different data sets (e.g. primary care, hospital, long term care facilities, mental health and social care) related to the same patient could facilitate not only calculation of indicators but also stratification algorithms to classify the patient according the level of complexity.

**8**	**eHealth**	Use eHealth and telemedicine technology with a clear medical objective to address individual patient needs. Facilitating virtual collaborative work and care between primary care and secondary care can be supportive.
**9**	**Fairness**	Chronic Care Management must practice fair collaboration, concepts, processes and strategies between all health model related stakeholders with focus on individual patient needs.
**10**	**Business**	A chronic care business model must be sustainable and address patient needs. Monitoring of treatment targets and cost-effectiveness should be part of the business considerations.
**11**	**Evaluation**	Implement measures for evaluation of the efficacy, effectiveness, quality and feasibility of the model. Predefined *Shared Outcome Frameworks* to regularly evaluate outcome measures without being limited to medical outcomes will anchor evaluation in individualised chronic care.
