# Supplementary material for: The Manage Care Model – Developing an Evidence-Based and Expert-Driven Chronic Care Management Model for Patients with Diabetes
Source: Int J Integr Care. 2020 Apr 22;20(2):2. doi: 10.5334/ijic.4646 (PMC7181948; doi:10.5334/ijic.4646)
Supplement: Annex 3. — Tables 4 and 5: Dimensions, sub-dimensions and examples derived from the literature review. [file ijic-20-2-4646-s3.pdf]

Annex 3

Table 4 Dimensions, sub-dimensions and examples derived from the literature review

**Dimensions**	1. Patient with type-2-diabetes (PWD)	2. Self-Management support	3. HCP/Clinical and professional leadership and commitment	4. Living environment	5. Broad community	6. National health and social system
**Sub-dimensions**	none					
**Examples (limited to 5)**	Well educated regarding own health, diagnosis,diseases and course of disease	Raising the empowerment of the patient by education and knowledge	Well educated professionals (medical knowledge proactive, prepared and foresighted)	Support in managing patients´ condition	Population-based health care planning	Health system infrastructure – Availability of health professionals, emergency rooms and hospital beds
	Awareness of determinants of health, complications,risk factors, severity of disease and disease itself	Building health literacy	24/7 coverage especially guaranteeing out-of-hours services	Cultural competency	Map of community resources available and accessible to citizens	Joined-up collaboration between health professionals and health sectors
	Improve self-management skills to manage own disease and plan their care, support and treatment(behavior, knowledge, motivation)	Support self-management skills (behavior, knowledge, motivation)	Experience of care - Expertise in medical field	Socioeconomic factors (e.g. minorities)	Accessible early detection of diseases and diagnosis of multimorbidity	Integrative approach of care including palliative care
	Well educated through self-management education programs to improve health	Educational and tailored training programs	Joined-up collaboration/Interdisciplinary workforce between health professionals and	Patient safety	Screening and disease prediction tools	Hospice-hospital partnerships and long-term care

	literacy (health education)		health sectors			
	Acceptance of own illness	Focus on glucose- and cardio-vascular-orientated secondary- and tertiary prevention	Sector of quality management for health care delivery	Working groups/ Self-help groups	Prevention of diseases	Population based health planning
	Targeted activation	Focus on health promotion	Cooperation between health care and social care - Integrated health and social care	Effective local area partnerships	Rehabilitation + Reintegration	General population stratification incorporating data from hospital and primary care minimum data sets
	Individual care plan and an allocated named professional (known single point of contact) who oversees their case	Create accessible knowledge (database)	Importance of gatekeeping role (GP and community nursing) although with a collaborative approach	Care Coordination outside the practice (e.g. transportation to appointments) through help groups	Implementation of diabetescenters	Healthy public policy environment - Supportive organizational, governance and leadership structures
	Shared decision making (SDM) and partnership with health professionals/provider of care allows individual care plans and goal-setting agreements between doctors and patients	Shared decision making (SDM) and partnership with health professionals/provider of care allows individual care plans and goal-setting agreements between doctors and patients	Shared decision making (SDM) and partnership with health professionals/provider of care allows individual care plans and goal-setting agreements between doctors and patients	Integrated, community-based care	Enable and check availability, accessibility, affordability and equity of care	Supportive, proactive and preventive insurances
	Transitional care: discharge planning and post-discharge support for people returning home after	Patient-centered care	support the whole “living with diabetes” (own and family knowledge, social	available technical aids and home adaptationssupporting safety at home	Build regional health care networks	state programs to cover the cost of health care for uninsured population

	hospital		aspects…)				
	Generating an advanced chronic disease model, related to patients requiring palliative orientation with advanced chronic diseases	Quality-materials recommendations available: apps, written materials, videos	Development of an education and training structure, In-service training			Development of standards and principles for high quality care	Supportive payments (e.g. giving local councils and authorities financial support to realize integrative care)
		Expert Patient Program initiatives (peer2peer)	Care Coordination: accountable professionals (care/case manager) and accountable team			Evidence-based, comprehensive and populations-based care using practice guidelines	Evaluated towards an Integrated Health and social care model
			Leadership involvement and commitment to construct pathways and local agreements			Integrated, community-based care	Pay-for-performance

Table 5 Dimensions, sub-dimensions and examples derived from the literature review (continued)

**Dimensions**	7. Care Delivery Strategy						8. Stratification and predictive modeling	9. Joint and shared Outcome Framework
**Sub-dimensions**	Care delivery management	Clinical management	Referral and Discharge management/Transitional Care	Health Data and Information management	Financial management	Quality management	none	
**Examples**	24/7 availability in case of crisis	Frequency/intensity of contacts hospital-health care team/ provider	Qualification of Case managers	Risk communication tools	Remuneration	Quality manual	To identify people at high(er) risk	Outcomes agreed and used by Primary Health Care, Community Nurses clinics, and Outpatient care in Policlinics
	Ensure integrative and joined-up delivery of care	Evidence-based diagnostic and treatment	Implemenatation and use of consolidated referral pathways including discharge planning, post-discharge support	Availability of data information systems enabling comparisons across providers, communities and even with other countries	Transforming payment models to incentative integrated care approach and to a joint and shared Outcome Framework	Performance measurement/ assessments	probability ofincreased utilisation of services (especially regarding emergency admissions related to chronic conditions)	
	Practice and delivery redesign: care	Integrated Care pathways incorporating also Social	Discharge management following the established	Information Systems with high level of interoperability	Pay-for-performance/ Capita payment/	Evaluation	Assessment of risk/ Stratification of risk - Morbidity and	

	coordination - accountable professionals (care/case manager) and accountable team	Services, apart from Primary Care and Secondary Care and A&E services for the 24/7 model	steps of the case management process	between Primary Care, Hospital, Mental Health and Long Term facilities, sometimes working with the same patients	Morbidity-adjusted capita financing/ pay-for-quality		Multimorbidity group assignment and risk assessment producing risk scores	
	Computerized practice	Realize smooth transitions between different care settings		Integrative care - Information and Technology System provides “virtual work” between primary health care (GP and home care) and specialist	Transparency of total clearing items	Value-based purchasing (Reward for the grade of performance on a set of quality measures and the grade of improvement in performance relative to a baseline)		
	Equitable, easy and timely access to optimal care	Development of standards and principles for high quality care		Analytics should be based on aggregating different Minimum Data Sets provided from Primary Care, Hospital, long-term care facilities, mental health	Physicians quality reporting (financial incentives to physicians for reporting quality data)	Identification of critical factors for success; problems		

				and social services				
	Evidence-based screening procedures	Making sure that old people returning home after hospital have a temporary care plan			National health systems support local councils and authorities	Physicians and other clinicians' quality reporting (financial incentives to physicians for reporting quality data)		
	Using the potential of telehealth and telemedicine to improve communication of professionals	Evidence-based, comprehensive and populations-based care using practice guidelines				Audits		
	Long-term monitoring of patients	Qualification of HCPs				(External) Scientific evaluations		
	Support of transitional care process by providing temporary care plans	Internal audits: Check of daily routines and procedures for efficacy, state of the art and guideline adherence				Panel management in-reach tools to identify gaps of care, Panel management out-reach tools to reach patients with gaps of care		
	Case management:					Quality assurance		

	To incorporate Community Nurses working in very comprehensive and community-oriented model					strategies: a. Measured on patient-reported outcomes<br>b. Integrated care orientation contracts with common goals and targets among providers (primary care, hospital care),<br>c. Monitoring, Audits, Assessments		
	Appointment system, recall system (“eRecall”)							
	Ensure timely follow-up care (register, recall systems) - e.g. 48-hours Follow-up-Toolkit							
	Accessible electronic health record							
